# Sanitation and Superstition

**Published:** 1897-07-10

**Authors:** 


The Hospital

A Journal of

The Medical Sciences and Hospital Administration.

Vol. XXII.—No. 563.] [July 10, 1897.

### Sanitation and Superstition.

The complaints which have reached England with reference to the enforcement of certain life-saving precautions upon the population of the more crowded parts of Bombay, and of other Indian cities, and the assertion that this enforcement has been the underlying cause of the recent disturbances at Poona and at Chitpur, may be said to have been practically disposed of on Monday by the replies given by Lord George Hamilton to questions in the House of Commons. From those replies it would appear that Lord Sandhurst, not only by the general character of his instructions, but also by the personal care which he has devoted to the supervision of the necessary proceedings, has rendered it almost impossible for the allegations made by the complainants to be true. We have had an opportunity of hearing details of the work done at Bombay, details privately furnished by an English medical lady who was among those concerned in its accomplishment, and, from her report, although it was sometimes necessary to unroof dwellings in order to prevent them from being occupied, nothing was omitted that the most constant solicitude for the religious beliefs of the natives, or for the opinions which form the basis of their domestic habits, could be calculated to suggest. We know, on the authority of Mr. Rudyard Kipling, that "Pagett, M.P., was a liar, and a fluent liar therewith;" but even Pagett, whatever may be his accomplishments in this direction, need not scorn to sit at the feet even of a very ordinary native, and to accept him as a Gamaliel of mendacity. Any number of such documents as the Deccan Subha memorial could be manufactured in India on the shortest notice and at the smallest cost; and, although those who have followed Sir William Wedderburn's career are not likely to retain much belief in his discretion, they will not deny him the merit of courage for his willingness to discuss such a production in the House of Commons. It is none the less satisfactory, we need hardly say, that no opportunity of becoming the mouthpiece of native sedition is now likely to be afforded to him. Many changes have occurred in India since the Mutiny; and one of them is the increase in the opportunities possessed by official personages of knowing the thoughts, aspirations, and feelings of all classes of the natives. Even if the Governor of Bombay were likely to be misinformed by his *entourage*, no such liability would exist in the case of Sir M. Bhownaggree, whose emphatic denial of the statements of the Indian malcontents has lately been communicated to the *Times*. For the outbreak at Poona sanitation seems to have been merely an excuse put forward to conceal the causes to which it was really due; while that at Chitpur turns out to have been a squabble between Hindoos and Mahomedans, a sort of Orientalised version of incidents which have not been unknown when mobs professing different religions have chanced to encounter one another even in an island of the West.

There can be no doubt, nevertheless, that the natives of India do not like improved sanitation, and that the duty of insisting upon it is one which, like many other of our duties in the country, will task at once the energies and the sagacity of those who represent the ruling power. Perhaps, the most notable effect of British domination has been the increase of population naturally arising from security for life and property and from the development of various forms of industry. Increase of population alone, without other changes, always brings with it an increased liability to disease; and this effect has been intensified in some cities, and very conspicuously in Bombay, by the introduction of the factory system, and by the overcrowding of enormous buildings which have been erected for the reception of the "hands." We cannot allow the famines and pestilences which served as a check to numbers under native rule to exert their sway under ours; and we must gradually educate the people to accept protection against disease at our hands, as they have already accepted protection against anarchy, against robbery, and against murder. Here and there protests will be made, or resistance will be offered; but the protests must be disregarded, and the resistance must be either evaded or overcome. Superstition is opposed to civilisation, and the waves of progress must ebb as well as flow. Notwithstanding the ebb, the tide will advance; and the results of some part of each day's labour will be permanent. Revolts, if they should arise, will be chiefly fomented by persons whose pecuniary interests are at stake—as by priests who live by offerings intended to propitiate malevolent deities—and such persons need not be very difficult to deal with. The great enemy to superstition is education in physical science; and, although a century must elapse before the effects of such education can percolate through the millions of India, no pains should be spared to bring it home to the minority by which the rudimentary public opinion of the masses is gradually moulded. When we think of the superstitions of the Greeks of the time of Thucydides, we shall see that the literary training of a Bengal Baboo may leave utterly untouched the terror of his childhood for "Kali," the wife of "Siva the Destroyer."

